# Auditory perception exhibits sexual dimorphism and left telencephalic dominance in *Xenopus laevis*

**DOI:** 10.1242/bio.035956

**Published:** 2018-12-15

**Authors:** Yanzhu Fan, Xizi Yue, Fei Xue, Jianguo Cui, Steven E. Brauth, Yezhong Tang, Guangzhan Fang

**Affiliations:** 1 Department of Herpetology, Chengdu Institute of Biology, Chinese Academy of Sciences, No.9 Section 4, Renmin South Road, Chengdu, Sichuan, People's Republic of China; 2 University of Chinese Academy of Sciences, 19A Yuquan Road, Beijing, People's Republic of China; 3 Sichuan Key Laboratory of Conservation Biology for Endangered Wildlife, Chengdu Research Base of Giant Panda Breeding, 26 Panda Road, Northern Suburb, Chengdu, Sichuan 610081, People's Republic of China; 4 Department of Psychology, University of Maryland, College Park, MD20742, USA

**Keywords:** Auditory perception, Sexual dimorphism, Telencephalon, Event-related potentials (ERPs), *Xenopus laevis*

## Abstract

Sex differences in both vocalization and auditory processing have been commonly found in vocal animals, although the underlying neural mechanisms associated with sexual dimorphism of auditory processing are not well understood. In this study we investigated whether auditory perception exhibits sexual dimorphism in *Xenopus laevis*. To do this we measured event-related potentials (ERPs) evoked by white noise (WN) and conspecific calls in the telencephalon, diencephalon and mesencephalon respectively. Results showed that (1) the N1 amplitudes evoked in the right telencephalon and right diencephalon of males by WN are significantly different from those evoked in females; (2) in males the N1 amplitudes evoked by conspecific calls are significantly different from those evoked by WN; (3) in females the N1 amplitude for the left mesencephalon was significantly lower than for other brain areas, while the P2 and P3 amplitudes for the right mesencephalon were the smallest; in contrast these amplitudes for the left mesencephalon were the smallest in males. These results suggest auditory perception is sexually dimorphic. Moreover, the amplitude of each ERP component (N1, P2 and P3) for the left telencephalon was the largest in females and/or males, suggesting that left telencephalic dominance exists for auditory perception in *Xenopus*.

## Abstract (precis)

**Summary:** Investigation of auditory neural mechanisms in the South African clawed frog (*Xenopus laevis*) indicates that auditory perception exhibits sexual dimorphism and left telencephalic advantage.

## INTRODUCTION

Distinct vocalizations are often used to convey multiple types of information about species, sex, reproductive status and location in vocal species including birds, insects and anuran amphibians (Gerhardt and Bee, 2006; Naguib et al., 2009; Tobias et al., 2010; Xu et al., 1996). Furthermore, vocal communication, including the production, transmission, perception and responses to these acoustic signals, is essential for both survival and reproductive success in these species (Kelley, 2004; Naguib et al., 2009; Vignal and Kelley, 2007). Accordingly, communication sounds typically exhibit distinctive spectral and/or temporal attributes that may vary among individuals or between sexes thus providing sources of information for the neural processes underlying species discrimination and individual recognition. Consistent with this, species employing vocal communication typically exhibit sexual dimorphism in their responses to conspecific or heterogenous vocalizations at the behavioral, electrophysiological and gene expression levels (Avey et al., 2008; Bernal et al., 2007; Pollack, 1982; Williams, 1985).

For example, male swamp sparrows (*Melospiza georgiana*) respond aggressively to synthetic songs made up of naturally occurring swamp sparrow syllables, even if these are presented in an uncharacteristic temporal sequence of swamp sparrows (Peters et al., 1980; Searcy et al., 1981a). Female swamp sparrows, however, preferentially respond with a soliciting display to songs made up of swamp sparrow syllables arranged in the species-specific temporal pattern (Searcy et al., 1981b). Similarly, anuran males are much more likely than females to respond to species-typical signals which show marked variation (Bernal et al., 2007). Moreover different call note types have been shown to convey separate messages to males and females in some anuran species (Wells and Schwartz, 2007). At the neural level, the significance of acoustic features can differ for males and females accompanied by sex differences in the sensitivity of neurons in the auditory periphery and midbrain in anurans (Narins and Capranica, 1976; Shen et al., 2011). For example, the sexes may differ in the frequency at which they are most sensitive, particularly at the high best frequency (HBF) of the basilar papilla (BP) (Keddy-Hector et al., 1992; McClelland et al., 1997; Narins and Capranica, 1976; Wilczynski et al., 1984). In addition, call stimulation induces significantly higher neuronal expression of the transcription factor ZENK compared to silence in the hippocampus and auditory forebrain areas (i.e. the caudomedial nidopallium and mesopallium) of female but not male zebra finch (*Taeniopygia guttata*) (Gobes et al., 2009). These results support the idea that male and female zebra finches show different patterns of neuronal activation in response to sexually dimorphic calls. Nevertheless, the prevalence of such sex differences in auditory processing and perception across land vertebrate species remains unclear. The present study focused on an anuran species, *Xenopus laevis*, insofar as all land vertebrates are derived from an amphibian stem.

In anurans, survival and reproductive behaviors depend primarily on a listener's ability to parse incoming sound signals that convey species identity and reproductive state (Bee, 2012). Anurans typically exhibit a small vocal repertoire and communicate in well-defined behavioral contexts making these species well suited for studies of acoustic signal perception and auditory system processing (Mangiamele and Burmeister, 2008; Mudry et al., 1977). For most anuran species, social behavior is generally characterized by sex differences in the production of vocalizations (Diekamp and Gerhardt, 1992). Under the classic paradigm, males are highly vocal and generally produce species-specific advertisement calls to attract females for breeding, as well as to deter rivals (Arch and Narins, 2009; Kelley, 2004; Tobias et al., 2010). In addition, females are often mute or possess a severely limited vocal repertoire with limited complexity (McClelland and Wilczynski, 1989; McClelland et al., 1997; Stewart and Rand, 1991). In some anuran species auditory tuning characteristics exhibit sexual dimorphism (Hall et al., 2016; Narins and Capranica, 1976; Wilczynski, 1986; Wilczynski and Capranica, 1984; Wilczynski et al., 1992), implying that sex differences could well exist in auditory perception in these species. However, very little is known or has been hypothesized about sexual dimorphism in central nervous system auditory processing.

The South African clawed frog, *X.* *laevis*, is a totally aquatic nocturnal species inhabiting silt-filled ponds that exhibits a relatively rich vocal repertoire (Tinsley and Kobel, 1996; Tobias et al., 2010; Vignal and Kelley, 2007). The frogs produce sexually distinctive calls based on simple click trains (trills) which differ in rate, temporal structure and intensity modulation for social communication (Brahic and Kelley, 2003; Rhodes et al., 2007). *Xenopus* males can produce six call types, the most prominent of which are advertisement calls produced during the breeding season to attract gravid females and compete with rivals (Mangiamele and Burmeister, 2008; Tobias et al., 2004). *Xenopus* *laevis* advertisement calls exhibit a bimodal temporal pattern consisting of fast (19 ms inter-click intervals) and slow (38 ms inter-click intervals) trill portions chained together in repetitive bouts (Elliott et al., 2007; Tobias et al., 2010), although it is still unclear which portion (the fast trill or slow trill) constitutes the initial period of the calls. *Xenopus laevis* females produce two types of calls, a sexually receptive rapping call that increases male vocal activity and an unreceptive ticking call that depresses male vocal activity (Tobias et al., 1998). For reproductive success both females and males must distinguish among these different signal variants (Andersson and Simmons, 2006; Vélez et al., 2013). Previous studies have showed that each male and female call type is distinguished by characteristic click rates (Vignal and Kelley, 2007). Thus males can use click rate to distinguish the sex of callers and discriminate between the female ticking and rapping calls (Elliott and Kelley, 2007; Vignal and Kelley, 2007). Consequently, click rate in particular plays a primary role for vocal communication in *X. laevis* (Elliott et al., 2011).

In anurans including *X. laevis*, auditory information derived from the tympanum is conveyed by the VIIIth nerve, which projects to the dorsal medullary nucleus (Edwards and Kelley, 2001; Elliott et al., 2011), which then projects to the laminar nucleus of the midbrain torus semicircularis (TS) (Edwards and Kelley, 2001; Kelley, 1980). From the midbrain, ascending auditory signals are conveyed to the central amygdala (CeA) in the telencephalon via the central thalamic nucleus (Hall et al., 2013). Both the TS and CeA are important brain areas for the temporal processing of auditory signals in *X. laevis* (Edwards and Kelley, 2001; Hall et al., 2013). Previous studies have demonstrated a high degree of sexual dimorphism for the central nervous system (CNS) vocal pathways, the larynx itself and the sensitivity of the peripheral auditory apparatus to species-specific frequencies in male advertisement calls in *X. laevis* (Hall et al., 2016; Kelley, 1986). For this reason we hypothesized that CNS processing for auditory perception is also sexually dimorphic in this species. Furthermore, it is well established that discrete brain regions are specialized for different functions (Kandel et al., 2013) and important neuroanatomical features of the brain have been conserved during vertebrate brain evolution (Finlay et al., 2001; Northcutt, 2002). Although the amphibian forebrain is not as well differentiated as that of amniotes (Feng and Schellart, 1999; Wilczynski, 1992; Wilczynski and Endepols, 2007), the striatum and the superficial and deep thalamic structures have been implicated in call recognition (Endepols et al., 2003) and electrophysiological studies have shown that communication sounds are processed preferentially in the left hemisphere in Emei music frogs (*Babina daunchina*) (Fang et al., 2014). Accordingly, we also hypothesized that left telencephalic dominance for auditory perception exists in *X. laevis*.

Event-related potentials (ERPs) are voltage fluctuations in the electroencephalogram (EEG) induced within the brain that are time locked to sensory, motor or cognitive events (Friedman et al., 2001). In this study ERPs were used to assess auditory processing in *X. laevis*, in midbrain and forebrain structures. To do this we recorded the EEG from telencephalic, diencephalic and mesencephalic sites and measured changes in three ERP components (N1, P2 and P3) evoked by three different stimuli, fast-slow trill calls (FS), slow-fast trill calls (SF) and white noise (WN), a stimulus lacking any of the temporal and spectral characteristics of the sounds produced by this species. Auditory ERPs provide a rich source of information about CNS information processing in structures activated by auditory stimuli, and research has shown that specific ERP components reflect specific aspects of auditory perception such as attention, stimulus categorization and the recognition of stimulus novelty (Näätänen and Winkler, 1999). Auditory ERPs are generally composed of three main components (N1, P2 and P3) which peak at latencies of ∼80 ms, ∼200 ms and ∼300 ms, respectively (Luck and Kappenman, 2011; Näätänen and Picton, 1987; Shahin et al., 2005; Tremblay et al., 2001; Tremblay and Kraus, 2002). Functionally, N1 is sensitive to selective attention (Näätänen and Picton, 1987), P2 reflects neural processes sensitive to the subject's familiarity with the acoustic stimulus and the specific acoustic features required to evaluate and classify stimuli previously experienced (Shahin et al., 2005), while P3 reflects brain activity corresponding to the psychological changes induced by the stimulation (Polich, 2011), also known as ‘novelty P300’ (Friedman et al., 2001). The present study measured the amplitudes and latencies of each ERP component for the left and right hemispheres in response to three acoustic stimuli (FS, SF and WN) for both sexes in order to investigate whether auditory perception is sexually dimorphic and to determine if left telencephalic dominance exists*.*

## RESULTS

**Fig. 1.**
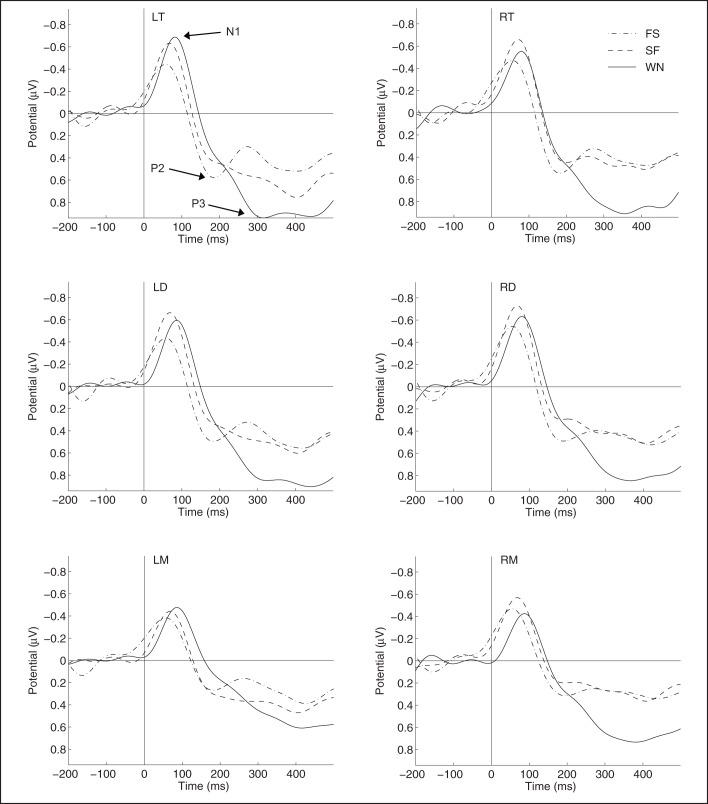
**Grand average waveforms for different brain regions during playbacks of WN, FS and SF calls, respectively (*n*=16).** Abbreviations: LT and RT, the left and right telencephalon; LD and RD, the left and right diencephalon; LM and RM, the left and right mesencephalon; WN, white noise; FS, fast-slow trill call; SF, slow-fast trill call.

The grand average of the original waveforms including N1, P2 and P3 are shown for each stimulus and each brain region in Fig. 1. There were significant differences among stimuli, brain areas and sexes in amplitude rather than latency for each ERP component, respectively.

### N1

**Fig. 2.**
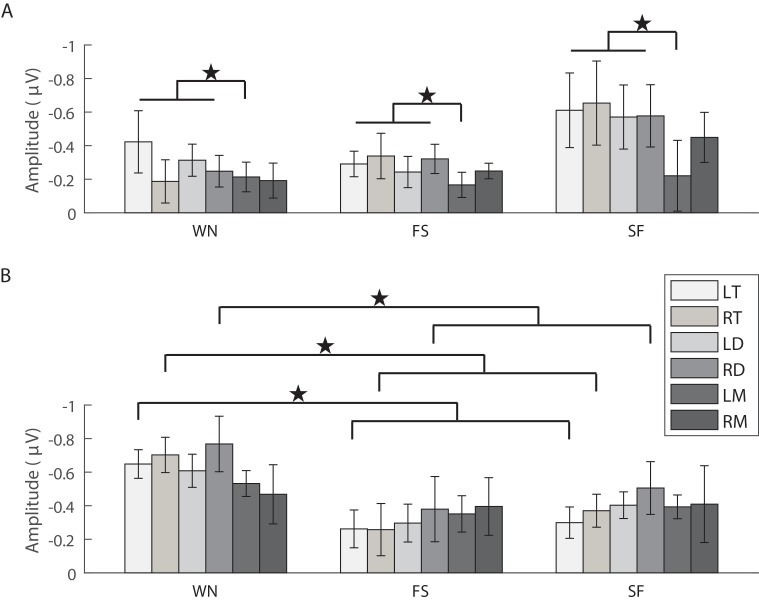
**Means and standard deviations for the N1 amplitudes evoked by each acoustic stimulus in each brain region for females (A) and males (B), respectively (*n*=16).** Filled stars denote that there were significant differences between different brain areas or different acoustic stimuli (*P*<0.05). Abbreviations: LT and RT, the left and right telencephalon; LD and RD, the left and right diencephalon; LM and RM, the left and right mesencephalon; WN, white noise; FS, fast-slow trill call; SF, slow-fast trill call.

The analysis for the N1 amplitude showed that there was no significant main effect for the factors ‘sex’ (*F*_1,14_=0.782, partial *η*^2^=0.053, *P*=0.391), ‘stimulus’ (*F*_2,28_=1.529, *ε*=0.968, partial *η*^2^=0.098, *P*=0.234) and ‘brain area’ (*F*_5,70_=1.031, *ε*=0.520, partial *η*^2^=0.069, *P*=0.383). However, the interaction among these three factors was significant (*F*_10,140_=2.118, partial *η*^2^=0.131, *P*=0.027). Simple and simple-simple effects analysis showed that the N1 amplitude in the left mesencephalon for females was significantly lower than those in the both sides of telencephalon and diencephalon (*F*_5,35_=3.799, *ε*=0.708, partial *η*^2^=0.352, *P*=0.007; Table 1 and Fig. 2A). The N1 amplitude evoked by WN for males was significantly higher than those for females (*F*_1,14_=8.423, partial *η*^2^=0.376, *P*=0.012; Table 1), particularly in the right telencephalon (*t*_14_=3.098, Cohen's *d*=1.656, *P*=0.008) and the right diencephalon (*t*_14_=2.731, Cohen's *d*=1.460, *P*=0.016). For males, the N1 amplitude evoked by WN was significantly larger than those evoked by conspecific advertisement calls (FS and SF calls) in both sides of telencephalon (*F*_2,14_=8.801, *ε*=0.769, partial *η*^2^=0.557, *P*=0.003 for the left telencephalon; *F*_2,14_=6.818, *ε*=0.777, partial *η*^2^=0.493, *P*=0.009 for the right telencephalon) and the right diencephalon (*F*_2,14_=5.426, *ε*=0.887, partial *η*^2^=0.437, *P*=0.018) (Table 1 and Fig. 2B).

**Table 1.**
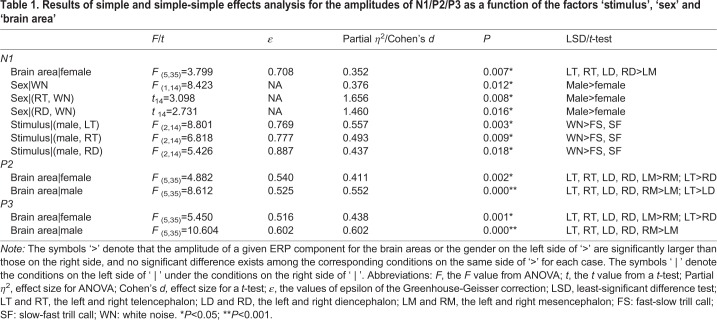
**Results of simple and simple-simple effects analysis for the amplitudes of N1/P2/P3 as a function of the factors ‘stimulus’, ‘sex’ and ‘brain area’**

In addition, for N1 latency there was no significant main effect for the factors ‘sex’ (*F*_1,14_=0.001, partial *η*^2^=0.000, *P*=0.981), ‘stimulus’ (*F*_2,28_=1.624, *ε*=0.776, partial *η*^2^=0.104, *P*=0.215) and ‘brain area’ (*F*_5,70_=2.193, *ε*=0.707, partial *η*^2^=0.135, *P*=0.065), however, the interaction between ‘sex’ and ‘brain area’ was significant (*F*_5,70_=3.125, partial *η*^2^=0.182, *P*=0.013). Simple effects analysis showed that the N1 latency in the left diencephalon for females was significantly longer than that of males (*t*_14_=2.361, Cohen's *d*=1.181, *P*=0.033). For males, the latency in the right mesencephalon was significantly longer than those in the left telencephalon and the right diencephalon (*F*_5,35_=3.003, *ε*=0.486, partial *η*^2^=0.300, *P*=0.023).

### P2

**Fig. 3.**
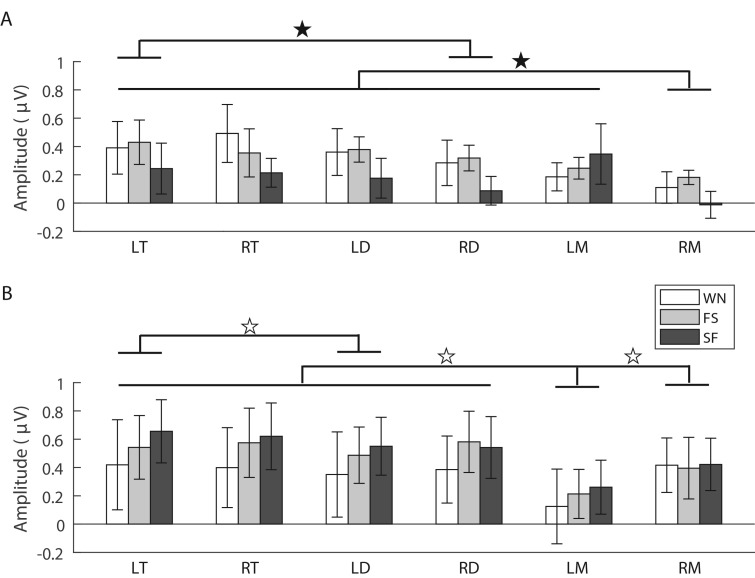
**Means and standard deviations for the P2 amplitudes evoked by each acoustic stimulus in each brain region for females (A) and males (B), respectively (*n*=16).** Filled stars and open stars denote that there were significant (*P*<0.05) and extremely significant (*P*<0.001) differences between different brain areas, respectively. Abbreviations: LT and RT, the left and right telencephalon; LD and RD, the left and right diencephalon; LM and RM, the left and right mesencephalon; WN, white noise; FS, fast-slow trill call; SF, slow-fast trill call.

The analysis for P2 amplitude showed that there was a significant main effect for the factor ‘brain area’ (*F*_5,70_=8.976, *ε*=0.645, partial *η*^2^=0.391, *P*=0.000) but not the factors ‘stimulus’ (*F*_2,28_=0.188, *ε*=0.742, partial *η*^2^=0.013, *P*=0.829) and ‘sex’ (*F*_1,14_=0.630, partial *η*^2^=0.043, *P*=0.441). In addition, the interaction between ‘sex’ and ‘brain area’ was significant (*F*_5,70_=4.430, partial *η*^2^=0.240, *P*=0.001). For females, P2 amplitude in the right mesencephalon was significantly lower than those in the other brain areas, while the P2 amplitude in the left telencephalon was significantly higher than that in the right diencephalon (*F*_5,35_=4.882, *ε*=0.540, partial *η*^2^=0.411, *P*=0.002; Table 1 and Fig. 3A). For males, the P2 amplitude in the left mesencephalon was significantly lower than in the other brain areas, while the P2 amplitude in the left telencephalon was significantly higher than that in the left diencephalon (*F*_5,35_=8.612, *ε*=0.525, partial *η*^2^=0.552, *P*=0.000; Table 1 and Fig. 3B).

In addition, for P2 latency there was no significant main effect for the factors ‘sex’ (*F*_1,14_=0.848, partial *η*^2^=0.057, *P*=0.373), ‘stimulus’ (*F*_2,28_=0.810, *ε*=0.788, partial *η*^2^=0.055, *P*=0.455) and ‘brain area’ (*F*_5,70_=1.052, *ε*=0.719, partial *η*^2^=0.070, *P*=0.394,), however, the interaction between ‘sex’ and ‘brain area’ was significant (*F*_5,70_=2.566, partial *η*^2^=0.155, *P*=0.034). Simple effects analysis showed that the latency in the right telencephalon for females was significantly longer than for males (*t*_14_=2.596, Cohen's *d*=1.299, *P*=0.021).

### P3

**Fig. 4.**
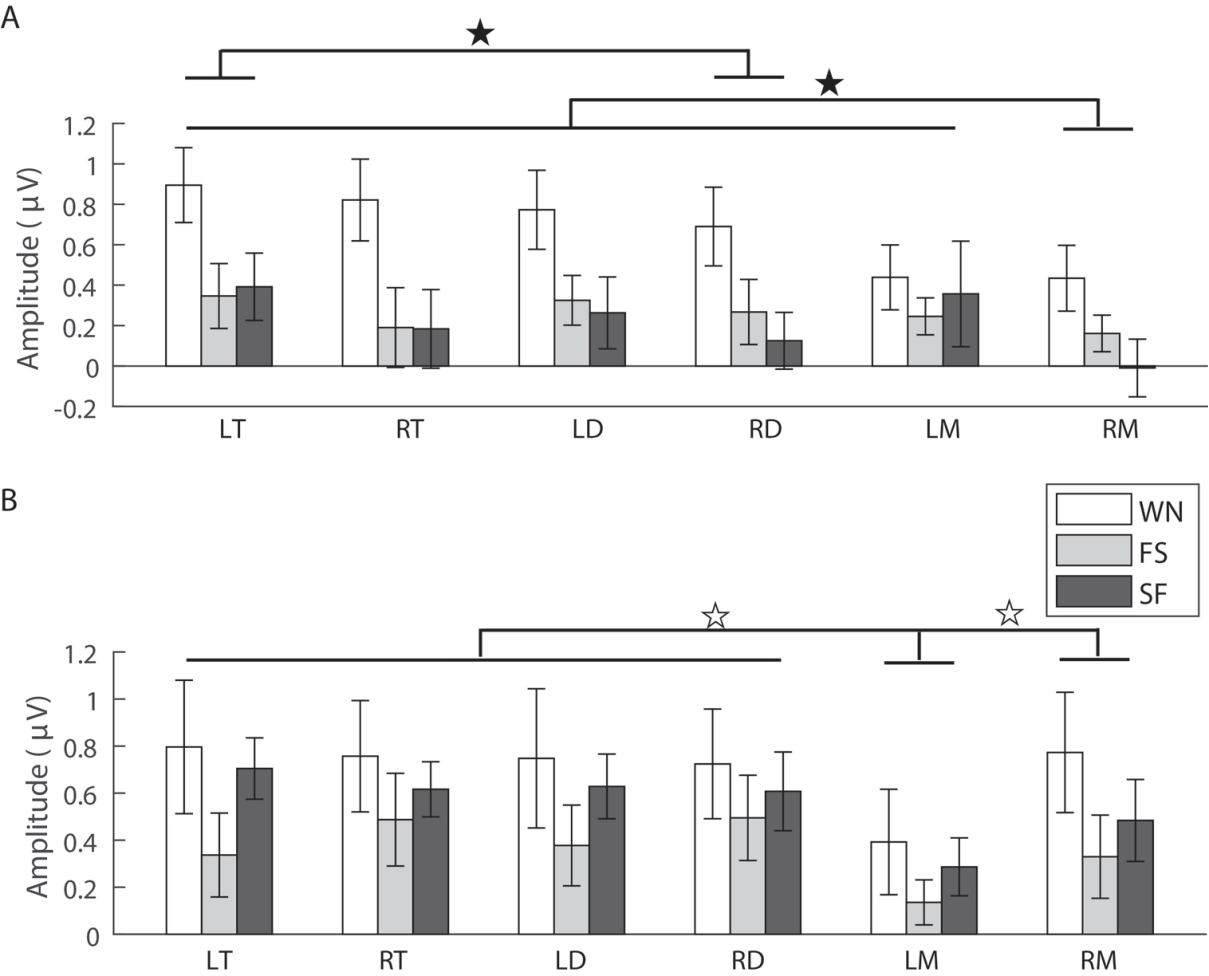
**Means and standard deviations for the P3 amplitudes evoked by each acoustic stimulus in each brain region for females (A) and males (B), respectively (*n*=16).** Filled stars and open stars denote that there were significant (*P*<0.05) and highly significant (*P*<0.001) differences between different brain areas, respectively. Abbreviations: LT and RT, the left and right telencephalon; LD and RD, the left and right diencephalon; LM and RM, the left and right mesencephalon; WN, white noise; FS, fast-slow trill call; SF, slow-fast trill call.

The analysis for the P3 amplitude showed that the main effects for the factors ‘stimulus’ (*F*_2,28_=4.908, *ε*=0.886, partial *η*^2^=0.260, *P*=0.015) and ‘brain area’ (*F*_5,70_=10.038, *ε*=0.670, partial *η*^2^=0.418, *P*=0.000) but not ‘sex’ (*F*_1,14_=0.653, partial *η*^2^=0.045, *P*=0.432) were significant. The P3 amplitude evoked by WN was significantly higher than those evoked by the FS and SF advertisement calls (Fig. 4A). The interaction between ‘brain area’ and ‘sex’ was significant (*F*_5,70_=5.041, partial *η*^2^=0.265, *P*=0.001). Simple effects analysis showed that the P3 amplitude in the right mesencephalon for females was significantly lower than those in the other brain areas. Furthermore, P3 amplitude in the left telencephalon was significantly higher than in the right diencephalon (*F*_5,35_=5.450, *ε*=0.516, partial *η*^2^=0.438, *P*=0.001; Table 1 and Fig. 4A). For males, the P3 amplitude in the left mesencephalon was significantly lower than those in the other brain areas (*F*_5,35_=10.604, *ε*=0.602, partial *η*^2^=0.602, *P*=0.000; Table 1 and Fig. 4B). In addition, for P3 latency there was no significant main effect for the factors ‘sex’ (*F*_1,14_=0.206, partial *η*^2^=0.014, *P*=0.657), ‘stimulus’ (*F*_2,28_=1.427, *ε*=0.723, partial *η*^2^=0.093, *P*=0.258) and ‘brain area’ (*F*_5,70_=1.289, *ε*=0.732, partial *η*^2^=0.084, *P*=0.279), and there was no interaction among these factors.

## DISCUSSION

The present study shows that when three stimuli consisting of WN, FS and SF calls are presented with equal probability (1) the amplitudes of N1 evoked in males by the WN stimulus for the right telencephalon and right diencephalon are significantly different from those evoked in females; (2) in males the N1 amplitudes evoked by conspecific calls are significantly different from that evoked by the synthesized WN stimulus while no difference obtained between FS and SF calls; (3) in females the N1 amplitude for the left mesencephalon was significantly lower than for other brain areas, while the P2 and P3 amplitudes for the right mesencephalon were the smallest. In contrast these amplitudes for the left mesencephalon were the smallest in males. These results are consistent with the hypothesis that neural processing for auditory perception is sexually dimorphic.

### Sexual dimorphism in auditory perception

In anurans, sex differences in acoustic communication are widespread (Hoke et al., 2008; Kelley, 1986, 2004; Liu et al., 2014). At the behavioral level, different types of call notes can convey separate messages to males and females in some anuran species (Wells and Schwartz, 2007). For this reason, males and females often react differently in response to conspecific calls. In addition, males are much more likely than females to respond to signals which vary from the species' norm (Bernal et al., 2007). These sexually dimorphic behaviors depend on neural systems that are sex-specific or common to males and females and potentially regulate a number of behaviors (Hoke et al., 2010). For example, in three *Xenopus* species (*X. amieti*, *X. petersii* and *X. laevis*), peripheral auditory sensitivity to the two dominant frequencies of conspecific male advertisement call is enhanced in females compared to males, while males are most sensitive to lower frequencies including those in the male-directed release calls (Hall et al., 2016). Furthermore, the sensitivity to these spectral features is modulated by ovarian signals in female *X. laevis*.

In the present study, WN served as a proxy for non-vocal signals which differ substantially from conspecific signals. N1 amplitudes in the right forebrain evoked by WN were significantly higher in males than in females. Moreover, N1 amplitude evoked by WN was significantly higher in males than those evoked by conspecific calls. These results are consistent with the idea that the negative N1 waves are affected by selective attention which enhances the perception of high-priority stimuli at the expense of other stimuli in the environment (Näätänen and Picton, 1987; Woldorff et al., 1993). Male *X. laevis* frogs produce advertisement calls to attract mates and compete with other males in the breeding season (Tobias et al., 2004), while females in the reproductive state are often silent or respond less to males (Tobias et al., 1998). Thus males would be more easily detected by predators compared to females. Animals generally maintain alertness to non-conspecific sounds which may be associated with danger (Haff and Magrath, 2010). Accordingly this strong selective pressure would likely result in a larger ‘N1 effect of selective attention’ (Hillyard et al., 1973) in males compared to females. Furthermore, for anurans, the sexes do differ in sensitivity at audiogram best frequencies, with males more sensitive in the lower frequency range (Hall et al., 2016; Miranda and Wilczynski, 2009). Females are more sensitive than males in response to natural vocalizations, despite showing no difference in response to pure tones at the same frequencies found within advertisement calls. However, thresholds to frequencies outside the range of the male advertisement call are higher in females (Liu et al., 2014; Miranda and Wilczynski, 2009). In addition, N1 is known to be sensitive to onset parameters (Biermann and Heil, 2000) and is influenced more by synchronous activity induced by the temporal envelope of the stimuli than by the spectral content in humans (Shahin et al., 2005). Thus, it is reasonable to speculate that differences between the temporal-spectral characteristics of the onsets of WN and the conspecific calls might contribute to the current results.

Significant differences in N1 amplitudes between sexes in humans have been found mainly in the right forebrain. These results are consistent with the idea that an important attentional network including the temporoparietal cortex and inferior frontal cortex is largely lateralized to the right hemisphere and specialized for the detection of behaviorally relevant stimuli, particularly when they are salient or unexpected (Corbetta and Shulman, 2002; Nobre et al., 2004; Shulman et al., 2010). More broadly, the right telencephalon has been proposed to be the site for rapid control of attention modulation (Evans et al., 2000; Fang et al., 2015; Yamaguchi et al., 2000), enabling individuals to efficiently acquire or change targets. Thus, right hemisphere dominance for selective attention and target detection might be a primitive trait present in amphibia. However it is noteworthy that the present results differ from our previous study showing a sexually dimorphic lateralized attention modulation network in Emei music frogs using Granger causal connectivity analysis, i.e. Granger causal connections in the left telencephalon are stronger in males while those in the right telencephalon are stronger in females (Xue et al., 2018). This difference might reflect species differences.

The present results show that the N1 latency for the left diencephalon and the P2 latency for the right telencephalon in females were significantly longer than those in males. These results are consistent with the idea that the latencies of ERP components are always positively correlated with perceptual processing demands (Callaway and Halliday, 1982; Courchesne et al., 1985; Kutas et al., 1977; Luck, 2014; Magliero et al., 1984; Polich, 2007) and that females are typically more selective in mate choice due to their greater reproductive investment. In addition, in females both P2 and P3 amplitudes for the right mesencephalon were significantly lower than those for other brain areas, consistent with the idea that left-hemispheric dominance for conspecific communication sounds exists in many vertebrates including frogs (Fang et al., 2014; Xue et al., 2015). Surprisingly, in males both P2 and P3 amplitudes for the left mesencephalon were the lowest. Future research is needed to determine the cause of this sexual dimorphism.

### The left telencephalon may play an important role in auditory perception

The present results show that the amplitude of each ERP component (N1, P2 and P3) for the left telencephalon are the largest of those for all other brain areas studied here in females and/or males. The P2 component reflects the process of signal evaluation and classification, and its amplitude can be enhanced by familiarity or similarity between the target and current stimulus (Bosnyak et al., 2004; Potts et al., 1998; Reinke et al., 2003; Shahin et al., 2005; Tremblay et al., 2010). While the P3 component can be elicited by a variety of stimuli, including those that are defined as targets as well as novels (Friedman et al., 2001; Novak et al., 1992), P3 amplitude is related to the cognitive processing demands of the eliciting stimulus (Anderer et al., 1998; Woodman, 2012). Thus the present results suggest that the left telencephalon plays an important role in auditory perception.

In amphibians, the midbrain torus semicircularis serves to relay brainstem auditory input to the forebrain as well as acting as a center for integrating ascending auditory and descending forebrain inputs (Endepols and Walkowiak, 2001; Wilczynski, 1981). The torus also acts as an audiomotor interface (Luksch and Walkowiak, 1998). Pathways originating in the midbrain give rise to ascending auditory input to the diencephalon and telencephalon, which is extraordinarily widespread. Nearly all of the telencephalon except specific olfactory areas receive some auditory input because the amphibian forebrain is relatively undifferentiated (Feng and Schellart, 1999; Wilczynski, 1992; Wilczynski and Endepols, 2007). For this reason there is no specific sensory area in the anuran telencephalon directly comparable to the auditory areas of the amniote telencephalon. Compared to amniotes the anuran pallium is not parcellated into discrete functional areas, although widespread connections linking forebrain neurons to motor and/or endocrine systems and limbic structures exist (Wilczynski and Endepols, 2007). However our knowledge about specific neuronal response properties in the anuran telencephalon is limited (Wilczynski and Endepols, 2007).

In contrast much is known about the role of the anuran midbrain in acoustic signal processing including *IEG* expression in response to conspecific calls (Burmeister et al., 2008; Wilczynski and Ryan, 2010). Studies have also reported *IEG* expression in multiple forebrain areas in response to conspecific calls (Hoke et al., 2007, 2010; Mangiamele and Burmeister, 2008). Ascending toral efferents gather in the lateral rostral torus and project to the ipsilateral diencephalon which consists of the thalamus dorsally and hypothalamus ventrally (Butler, 1995; Wilczynski and Endepols, 2007). Neurons in the posterior thalamic nucleus appear to be specialized for processing the temporal and spectral features of the species vocalizations, particularly advertisement calls (Wilczynski, 1992; Wilczynski and Endepols, 2007).

Previous studies have shown that two telencephalic areas, the striatum and medial pallium, receive ascending auditory input from the thalamus and contain neurons responsive to acoustic stimuli (Wilczynski and Endepols, 2007; Wilczynski and Ryan, 2010). Simple stimuli such as clicks generally fail to stimulate cells at this level of the CNS. In contrast, complex signals similar to naturally occurring calls can evoke large neuronal responses in the striatum and medial pallium (Wilczynski and Capranica, 1984). Moreover, lesions of the striatum and superficial and deep thalamic structures may disrupt vocal recognition (Endepols et al., 2003), suggesting that telencephalic areas play important roles in call recognition. Furthermore, we have previously shown that communication sounds are processed preferentially in the left hemisphere in Emei music frogs (Fang et al., 2014). Taken together with the present results in *X. laevis*, the accumulating evidence points to an important role for the left telencephalon in acoustic signal processing and auditory perception in anurans.

The present results show that the P3 amplitude evoked by WN was greater than the P3 amplitudes evoked by both conspecific call types, consistent with our previous finding showing that Emei music frogs classify conspecific calls into one category, and perceive WN as a novel stimulus (Fang et al., 2015). Typically the P3 component is most strongly elicited using oddball paradigms in which subjects are exposed to minor portions of unexpected/novel stimuli interspersed between major portions of uniform/standard stimuli (Friedman et al., 2001; Novak et al., 1992). However, the equiprobability paradigm used in the present study could produce an oddball-like paradigm in which the conspecific FS and SF calls could serve as the major stimulation (66.7% probability) while WN served as a minor novelty (33.3% probability). Thus, a larger P3 could have been evoked by WN because the animals, as would be expected, classified the two conspecific calls into one category.

## MATERIALS AND METHODS

### Animal

Experiments were performed on sixteen *X. laevis* frogs of both sexes (eight males and eight females) bred in our lab. The frogs were separated by sex and housed in two aquaria (120×50 cm and 60 cm deep) with a water depth of approximately 20 cm. The frogs were fed every three days and the water was replaced once a week. The aquaria were placed in a room under controlled temperature conditions (20±1°C) and maintained on a 12 h:12 h light–dark cycle (lights on at 08:00). The subjects measured 8.1±1.1 cm (mean±s.d.) in body length and 67.1±22.2 g in body mass at the time of surgery. All experimental procedures conformed to the requirements of the Animal Care and Use Committee of Chengdu Institute of Biology, Chinese Academy of Sciences.

### Surgery

All experiments were conducted during April to August, 2016 and June, 2017 (this species breeds between April and September in our lab), during which time calling in both sexes and a female oviposit were observed. Sexually mature adults of both sexes (males with nuptial pads, females with protruding cloacal labia) were chosen for the experiments (Hayes et al., 2010; Tobias et al., 2004; Van Wyk et al., 2003). The reproductive status of males was determined by recording call activity before and during the experiments and that of the females was determined by determining if each female was gravid after the experiments.

**Fig. 5.**
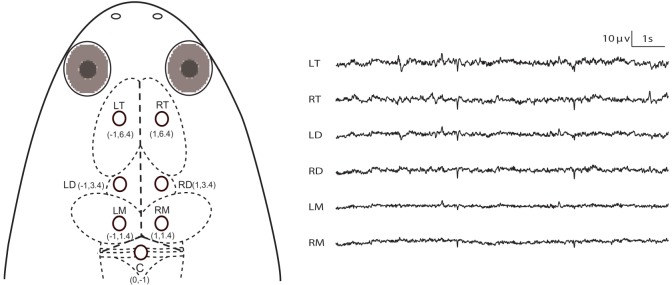
**Electrode placements and 10 s of typical EEG tracings for each channel.** The intersection of the three dashed lines in the head of *X. laevis* denotes the lambda (i.e. the vertex where skull sutures intersect). Abbreviations: LT and RT, the left and right telencephalon; LD and RD, the left and right diencephalon; LM and RM, the left and right mesencephalon. Image adapted from Fan et al. (2018) licensed under https://creativecommons.org/licenses/by/4.0/legalcode CC-BY 4.0 with permission.

Before surgery, the frogs were deeply anesthetized by immersion in a 0.35% solution of tricaine methanesulfonate (MS-222) and the optimum depth of anesthesia for surgery was determined by loss of the toe pinch response. After anesthesia, the frogs were covered with moist gauze to prevent dehydration. The skin and the underlying muscles of the operation area were cut away in order to expose the dorsal skull. Seven cortical EEG electrodes, consisting of miniature stainless steel screws (*φ* 0.8 mm), were screwed by turning through 3.5 circles to implant (about 1.1 mm deep) in the frog skull above the left and right sides of the telencephalon, diencephalon and mesencephalon (LT, RT, LD, RD, LM and RM). These electrodes were referenced to the electrode above the cerebellum (C) (Fig. 5). Ten seconds of typical EEG waves are presented along with the corresponding electrode pairs in Fig. 5. The electrodes above LT and RT were implanted bilaterally 6.4 mm anterior to the lambda (i.e. the vertex where the skull sutures intersect) and 1.0 mm lateral to the midline respectively, and the electrodes above LD and RD were implanted bilaterally 3.4 mm anterior to the lambda and 1.0 mm lateral to the midline respectively, while the electrodes above LM and RM were implanted bilaterally 1.4 mm anterior to the lambda and 1.0 mm lateral to the midline, respectively. The reference electrode (C) was implanted 1.0 mm posterior to the lambda at the midline (Fig. 5). One end of all electrode leads, formvar-insulated nichrome wires, was twined tightly on the screws and fixed on the skull of the frog with dental acrylic, while the other end was soldered to the pins of the light connector. If a frog regained motility during surgery, supplemental MS-222 solution was wiped onto the animal's skin using a cotton swab. Finally, the skin edges and muscles surrounding the wound were treated with an ointment with triple antibiotics and pain relief (CVS pharmacy, Woonsocket, USA) to prevent infection and discomfort. In addition, the connector was covered with self-sealing membrane (Parafilm®; Chicago, USA) for waterproofing.

After surgery, the frogs were covered with moist gauze and were placed on a sponge (17×10×1 cm^3^) which had absorbed about 200 ml of water. The sponge was placed at the bottom of a transparent plastic box with a floor area of 18×11 cm^2^ and 12 cm in height. After about 3 h, the frogs were recovered from anesthesia status and each frog was housed individually in an aquarium (34×24.5×18.5 cm^3^, with water depth approximately 10 cm) for 6 days for recovery before the experiments were performed. During the recovery period, the frog was fed every 3 days and the water replaced every day. After the end of all experiments, the subjects were euthanized by immersion in MS-222 solution for a prolonged period of time and peripheral blood leukocyte counts were acquired to verify that the animals were not infected during the experimental period (Hadji-Azimi et al., 1987) (Fig. S1; Table S1). Finally, the electrode locations were confirmed by injecting hematoxylin dye through the skull holes in which the electrodes had previously been installed (Fig. S2). In addition, the resistances between the reference and each of other electrodes were measured when the subjects were placed under water, while the corresponding resistances were also measured for a simulative electrode array under water in which the distances between every two electrodes were the same as those in the subjects. Since the resistances for the subjects were about 100 times as large as for the simulative electrode array, possible leakage water around the electrodes would be slight and could not influence the present results.

### Recording conditions

An aquarium (120×50 cm and 60 cm deep) with water depth of approximately 20 cm was placed in a soundproof and electromagnetically shielded chamber for which the background noise was 24.3±0.7 dB (mean±s.d.). In order to decrease sound reverberations which might be produced within the glass, the inner walls of the aquarium were covered by sound-absorbing cotton. The aquarium was separated into three equal parts by fine-mesh gauze. Light and temperature in the chamber were maintained as in the housing room. A video camera with an infrared light source and motion detector was appended centrally about 1 m above the middle part of the aquarium for monitoring the subject's behaviors. Electrophysiological signals were recorded with a signal acquisition system (Chengyi, RM6280C; Sichuan, China).

### Stimuli and procedure

**Fig. 6.**
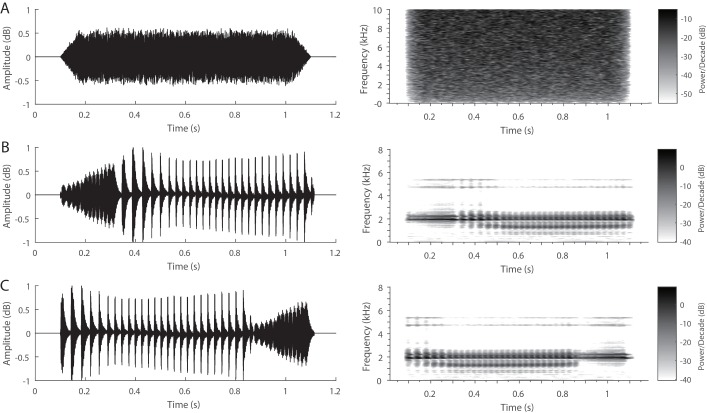
**Waveforms and spectrograms of the three stimuli for a randomly selected stimulus set.** (A) WN, white noise; (B) FS, fast-slow trill call; (C) SF, slow-fast trill call. Since the frequency response of the speaker is 0.1–10 kHz, white noise within the same bandwidth is shown.

The advertisement calls of *X. laevis* are biphasic and composed of alternating fast and slow trills (Elliott et al., 2007; Tobias et al., 2010). Previous work has shown that white noise (WN) removes the variability of stimulus novelty and can produce reliable ERP components (Combs and Polich, 2006; Frank et al., 2012; Yue et al., 2017). Accordingly, three types of stimuli were used: WN, FS (fast-slow trill call) and SF (slow-fast trill call, i.e. the reverse sequence of the FS stimulus). Since pseudoreplication may affect the conclusions of statistical analyses in ecological, animal behavior and neuroscience studies (Freeberg and Lucas, 2009; Kroodsma et al., 2001; Lazic, 2010; Schank and Koehnle, 2009), the possible effects of pseudoreplication were controlled by using multiple stimulus examplars in the present study. To do this, four advertisement calls of virtually identical durations were acquired from four different individuals by random selection from our dataset. Each call consisted of a fast trill and a slow trill (i.e. FS). The reverse versions of the four calls (i.e. SF) were acquired using simple cut and paste in Adobe Audition 3.0 software (San Jose, California, USA). The duration of the WN stimulus equaled the average duration of the four conspecific calls (about 1 s) and was shaped with rise and fall time sinusoidal periods of 50 ms (Fig. 6). Each set of stimuli was played back to four subjects using an equiprobability paradigm via two Daravoc underwater speakers (frequency response: 0.1–10 kHz; Sun Pride Inc., Zhejiang, China). The speakers were equidistantly placed at the bilateral sides of the testing aquarium. The root mean square (RMS) intensity of each stimulus was adjusted to be the same as that of a randomly selected call recorded in the experimental aquarium. Under these conditions, the sound level distribution at the bottom of the middle part of the aquarium was close to a quasi-free sound field. Furthermore, the subjects could move freely in this part of the aquarium throughout the experiments. Thus it is highly unlikely that the tiny differences in the stimulus amplitude across the tank bottom could have had a significant effect on the ERP measures. Since the influence of the target stimulus probability on P3 amplitude would weaken considerably under longer interstimulus intervals (Gonsalvez and Polich, 2002; Polich, 1987), the interstimulus interval was set to 1.5 s in the present study, the same as that used in a previous study on another frog species, the Emei music frog (Fang et al., 2015). For each subject, a total of 300 stimulus presentations with each stimulus presented 100 times were broadcasted in a random order. Randomization was constrained to prevent more than three stimuli from within the same acoustic category being presented successively.

Many studies have referred to the P3 ERP component as occurring mostly in oddball paradigms in which subjects are exposed to relatively rare unexpected/novel stimuli interspersed between major time portions consisting of a series of uniform/standard stimuli. It should therefore be noted that the equiprobability paradigm used in the present study could produce an oddball-like paradigm in which the conspecific FS and SF calls could serve as the major stimulation (66.7% probability) while WN served as a minor novelty (33.3% probability). In light of this, it would be expected that WN would evoke a larger P3 since the subjects would be expected to classify the two conspecific calls into one category.

### EEG signal acquisition and ERP analysis

After postoperative recovery for 6 days, the subject was placed in the middle of the experimental aquarium and connected to the signal acquisition system for about 2 h habituation. The connector was covered with a self-sealing membrane for waterproofing and waterproof adhesive was used when necessary. Then the EEG signal and behavioral data were collected according to the above described auditory stimulation paradigm.

To extract ERP components, EEG recordings were filtered offline using a band-pass filter at 0.25–25 Hz and a notch filter to eliminate possible interference at 50 Hz before averaging the stimulus-locked EEG epochs. The EEG signals were divided into epochs with a duration of 700 ms, including a prestimulus baseline of 200 ms. All single EEG trials were inspected visually and trials with muscle artifacts and electrode drifts were removed from all further analysis. Accepted trials were averaged according to stimulus types and brain areas within each session.

The auditory ERP component N1 was defined as the mean amplitude during latency intervals of 30–130 ms, P2 during intervals of 150–250 ms and P3 during intervals of 250–350 ms after stimulus onset (Luck and Kappenman, 2011; Näätänen and Picton, 1987; Shahin et al., 2005; Tremblay et al., 2001; Tremblay and Kraus, 2002). The latency was determined by the ‘50 percent area latency measure’ for each ERP component (Luck, 2014), i.e. measuring the area under the curve within the time windows and finding the time point that divided this area into equal halves. Both the amplitudes and latencies of the original waveforms were subjected to further statistical analyses for each ERP component.

### Statistical analyses

The normality of the distribution and the homogeneity of variance for ERP values were estimated with the Shapiro–Wilk *W* test and Levene's test, respectively. The amplitudes of N1, P2 and P3 were statistically analyzed using a four-way repeated measures ANOVA with the variables of ‘sex’ (male/female), ‘stimulus’ (FS/SF/WN), ‘brain area’ (LT/RT/LD/RD/LM/RM) and ‘stimulus set’ (the four stimulus sets). There was no significant main effect of the last factor for either the amplitude or the latency of each ERP component, consistent with the idea that the results of the present statistical analyses could not be affected by pseudoreplication. Thus, all data sets were pooled regardless of ‘stimulus set’ and statistically analyzed using a three-way repeated measures ANOVA including the first three factors. Both main effects and interactions were examined. For significant ANOVAs, data were further analyzed for multiple comparisons using the least-significant difference (LSD) test or *t*-test. Simple and simple-simple effects analysis were applied when the interaction was significant. Greenhouse-Geisser epsilon (*ε*) values were employed when the Greenhouse-Geisser correction was necessary. Effect size was determined with Cohen's d for *t*-tests and partial *η*^2^ for ANOVAs (partial *η*^2^=0.20 is a small effect size, 0.50 is a medium effect size and 0.80 is a large effect size). SPSS software (release 21) was utilized for the statistical analysis. A significance level of *P*<0.05 was used for all comparisons.

## Supplementary Material

Supplementary information
