# Supplementary material for: Auditory perception exhibits sexual dimorphism and left telencephalic dominance in Xenopus laevis
Source: Biol Open. 2018 Dec 15;7(12):bio035956. doi: 10.1242/bio.035956 (PMC6310876; doi:10.1242/bio.035956)
Supplement: Supplementary information [file biolopen-7-035956-s1.pdf]

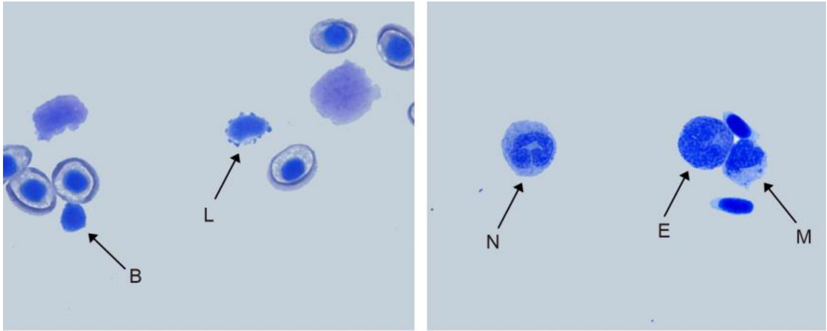

Figure S1. Peripheral blood smear stained with Rapid Wright-Giemsa stain. Abbreviations: B, Basophil; L, Lymphocyte; E, Eosinophil; M, Monocyte; N, Neutrophil.

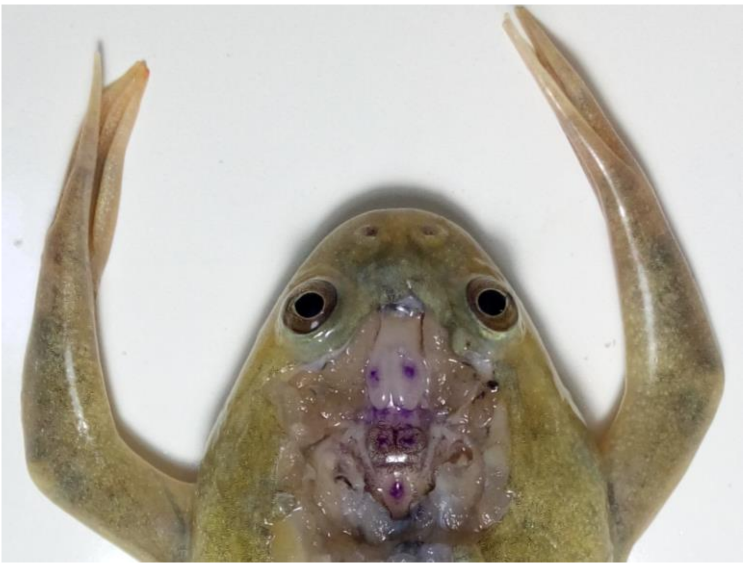

Figure S2. An photo of a typical preparation showing the positions of 7 electrodes on the skull of a subject. There were 2 electrodes above the telencephalon, diencephalon and mesencephalon respectively, while the reference was above the cerebellum.

Biology Open • Supplementary information

Table S1 Results of leukocyte counts in *X. laevis*

ID	Sex	Neutrophil	Eosinophil	Basophil	Monocyte	Lymphocyte
X.laevis 1	Male	22	5	46	3	24
X.laevis 2	Female	40	5	31	3	19
X.laevis 3	Male	32	2	44	1	21
X.laevis 4	Female	17	1	57	0	25
X.laevis 5	Male	39	2	42	2	15
X.laevis 6	Female	31	5	37	2	25
X.laevis 7	Female	32	4	38	3	23
X.laevis 8	Male	27	6	43	3	21
X.laevis 9	Male	36	5	39	2	18
X.laevis 10	Female	39	5	41	3	12
X.laevis 11	Female	21	2	58	3	16
X.laevis 12	Female	36	1	41	4	18
X.laevis 13	Female	31	3	41	2	23
X.laevis 14	Male	18	5	34	2	41
X.laevis 15	Male	26	2	51	0	21
X.laevis 16	Male	39	1	27	1	32
Average percentage for 16 animals ± SD		30.375±7.77	3.38±1.78	42.00±8.19	2.13±1.15	22.13±6.9
Normal range of percentage		17-40	1-6	27-58	0-4	12-41

Note: The normal range of the percentages of leukocyte counts is based on the reference "Hadji-Azimi, I., Coosemans, V. and Canicatti, C. (1987). Atlas of adult Xenopus *laevis laevis* hematology. *Developmental & Comparative Immunology* 11, 807-874.".
